# Author Correction: Bisphosphonate enhances TRAIL sensitivity to human osteosarcoma cells *via* death receptor 5 upregulation

**DOI:** 10.1038/s12276-018-0127-9

**Published:** 2018-08-17

**Authors:** Myung-Hee Moon, Jae-Kyo Jeong, Jae-Suk Seo, Jae-Won Seol, You-Jin Lee, Meilang Xue, Christopher J. Jackson, Sang-Youel Park

**Affiliations:** 1 Center for Healthcare Technology, Development Bio-Safety Research Institute, College of Veterinary Medicine, Chonbuk National University, Jeonju, 561-756 Korea; 2 Sutton Arthritis Research Laboratories, Institute of Bone and Joint Research, Kolling Institute, University of Sydney at Royal North Shore Hospital, St. Leonards, New South Wales Australia

**Correction to**: *Experimental & Molecular Medicine* (2011) **43(3)**, 138-145; 10.3858/emm.2011.43.3.016; Published Online: 7 February 2011

After publication of this article, the authors noticed an error in the figure section.

The correct figures of this article should have read as below.

**Fig. 2:**
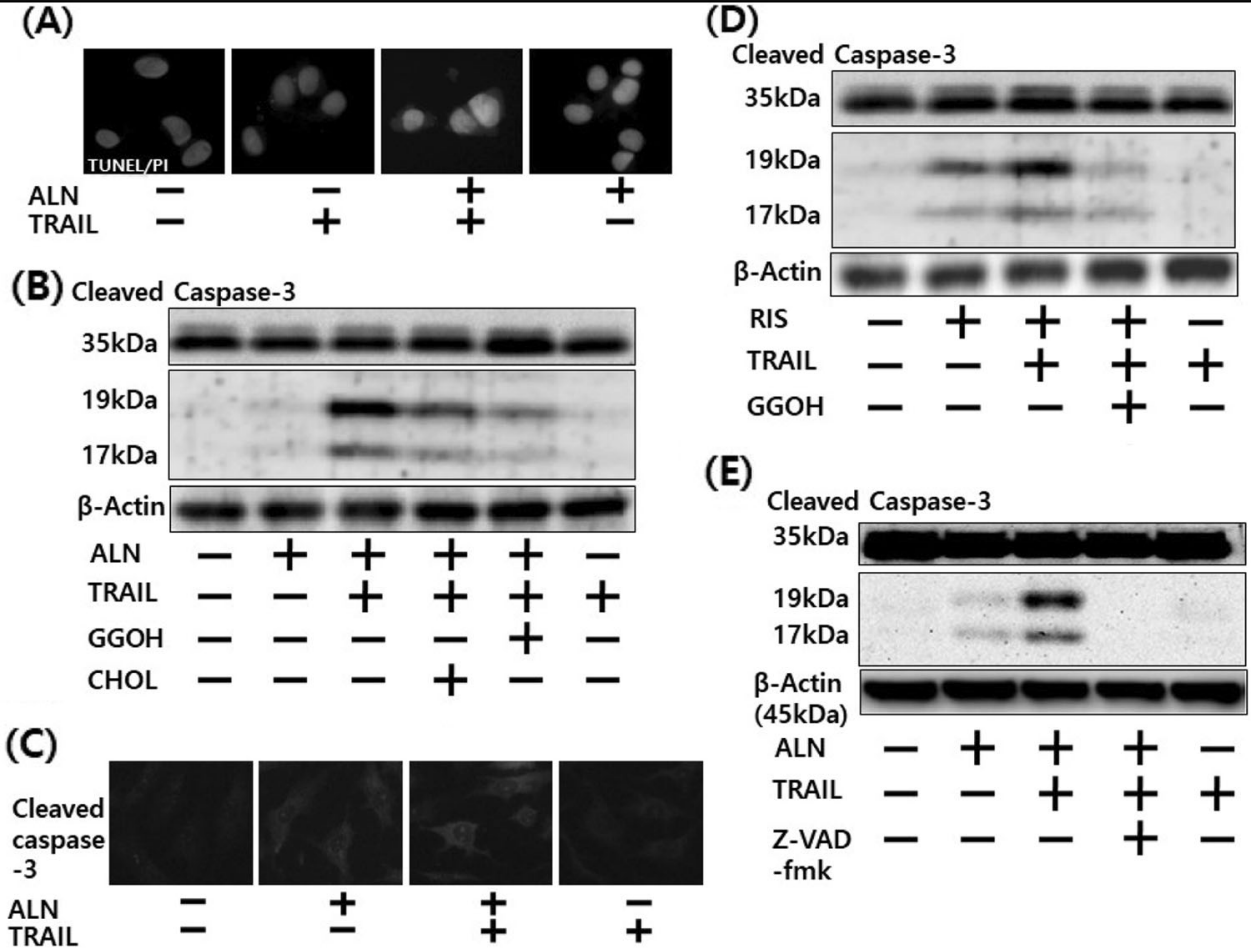
▓

**Fig. 3:**
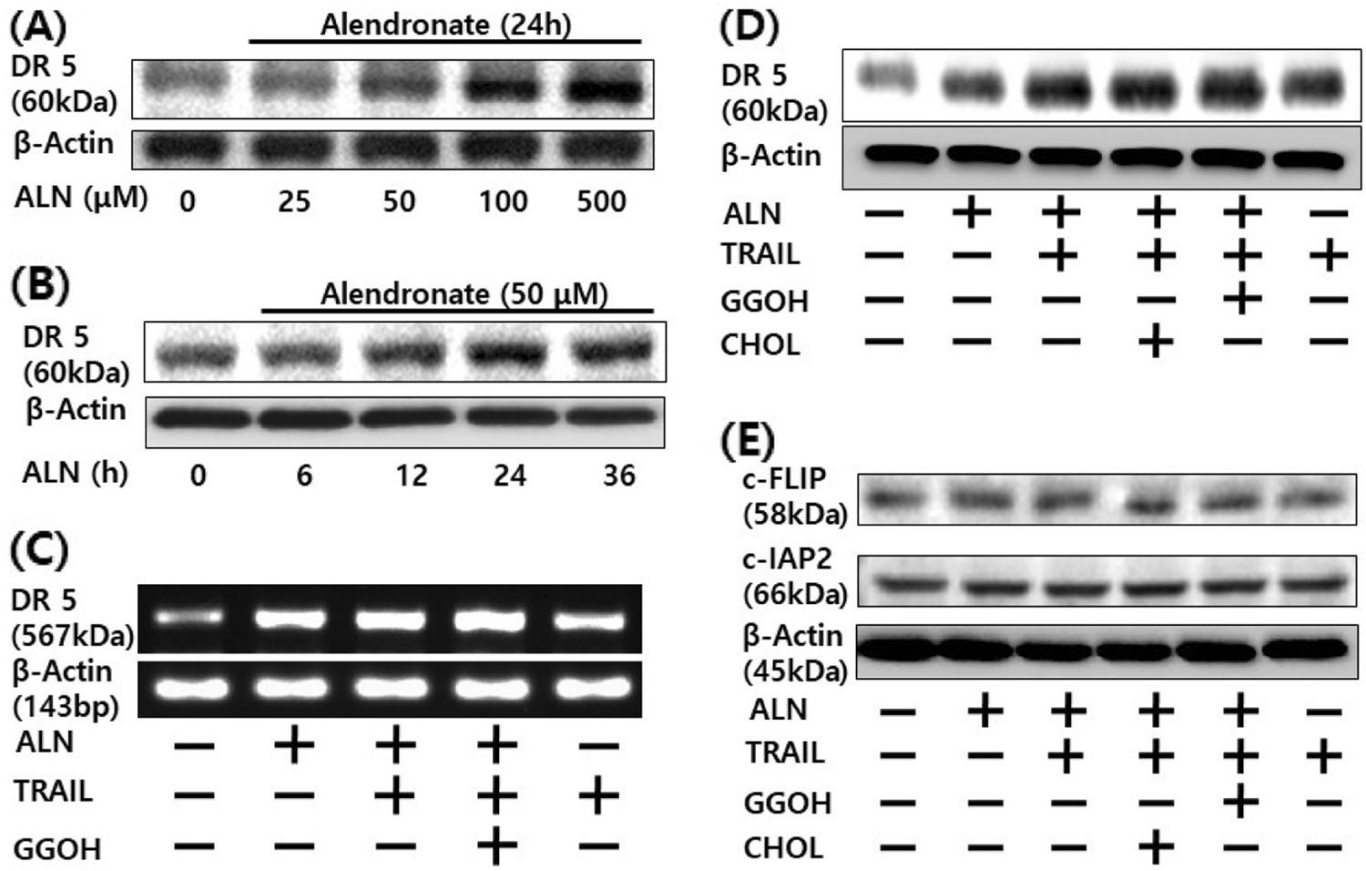
▓

**Fig. 4:**
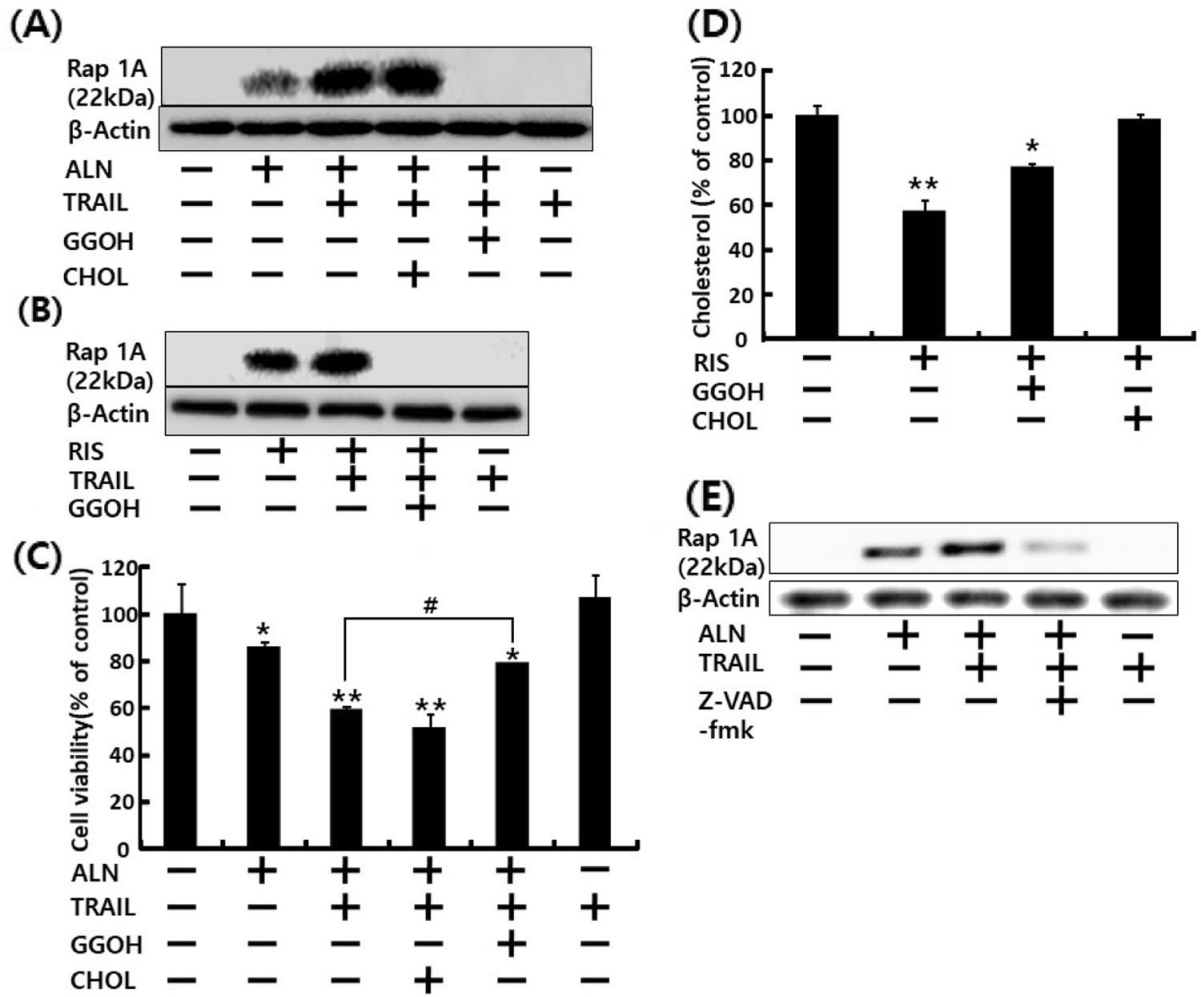
▓

The authors apologize for any inconvenience caused.

